# Aberrant functional connectivity in insular subregions in somatic depression: a resting-state fMRI study

**DOI:** 10.1186/s12888-022-03795-5

**Published:** 2022-02-24

**Authors:** Rui Yan, Ji Ting Geng, Ying Hong Huang, Hao Wen Zou, Xu Miao Wang, Yi Xia, Shuai Zhao, Zhi Lu Chen, Hongliang Zhou, Yu Chen, Zhi Jian Yao, Jia Bo Shi, Qing Lu

**Affiliations:** 1 Nanjing Brain Hospital, Medical School, Nanjing University, 22 Hankou Road, Nanjing, 210093 China; 2 Department of psychiatry, the Affiliated Brain Hospital of Nanjing Medical University, 264 Guangzhou Road, Nanjing, 210029 China; 3 Affiliated Mental Health Center & Hangzhou Seventh People’s Hospital, Zhejiang University School of Medicine, Hangzhou, China; 4 School of Biological Sciences and Medical Engineering, Southeast University, No. 2 sipailou, Nanjing, 210096 China; 5 Child Development and Learning Science, Key Laboratory of Ministry of Education, Nanjing, 210096 China

**Keywords:** Somatic depression, Ventral anterior insula, Dorsal anterior insula, Posterior insula, Resting-state functional connectivity

## Abstract

**Background:**

Somatic depression (SD) is different from non-somatic depression (NSD), and insular subregions have been associated with somatic symptoms. However, the pattern of damage in the insular subregions in SD remains unclear. The aim of this study was to use functional connectivity (FC) analyses to explore the bilateral ventral anterior insula (vAI), bilateral dorsal anterior insula (dAI), and bilateral posterior insula (PI) brain circuits in SD patients.

**Methods:**

The study included 28 SD patients, 30 NSD patients, and 30 matched healthy control (HC) subjects. All participants underwent 3.0 T resting state functional magnetic resonance imaging. FC analyses were used to explore synchronization between insular subregions and the whole brain in the context of depression with somatic symptoms. Pearson correlation analyses were performed to assess relationships between FC values in brain regions showing significant differences and the total and factor scores on the 17-item Hamilton Rating Scale for Depression (HAMD_17_).

**Results:**

Compared with the NSD group, the SD group showed significantly decreased FC between the left vAI and the right rectus gyrus, right fusiform gyrus, and right angular gyrus; between the right vAI and the right middle cingulate cortex, right precuneus, and right superior frontal gyrus; between the left dAI and the left fusiform gyrus; and between the right dAI and the left postcentral gyrus. Relative to the NSD group, the SD group exhibited increased FC between the left dAI and the left fusiform gyrus. There were no differences in FC between bilateral PI and any brain regions among the SD, NSD, and HC groups. Within the SD group, FC values between the left vAI and right rectus gyrus were positively correlated with cognitive impairment scores on the HAMD_17_; FC values between the right vAI and right superior frontal gyrus were positively related to the total scores and cognitive impairment scores on the HAMD_17_ (*p <* 0.05, uncorrected).

**Conclusions:**

Aberrant FC between the anterior insula and the frontal and limbic cortices may be one possible mechanism underlying SD.

**Supplementary Information:**

The online version contains supplementary material available at 10.1186/s12888-022-03795-5.

## Background

Major depressive disorder (MDD) is one of the most common mental disorders and is characterized by the presence of a depressive mood, loss of interest or pleasure, guilt and worthlessness, and somatic symptoms [1]. Somatic symptoms are common in patients with MDD [2]. Silverstein defined MDD patients with at least three somatic symptoms including fatigue, appetite, insomnia, unexplained breathing difficulty, poor body image, and pain, as having somatic depression (SD) [3]. Patients with SD are often misdiagnosed with other somatic diseases and repeatedly visit outpatient clinics of general hospitals [4]. Because of severe physical symptoms and poor curative effects, patients with SD are at high risk of suicidal ideation and poor quality of life [5].

Somatic symptoms are associated with greater clinical severity and lower remission rates, and pain symptoms have the greatest prognostic value [6]. Pain may be the sole complaint in SD patients who present to primary care practices and is often overlooked by clinicians [7]. The degree of pain is related to MDD severity, and is closely related to other somatic symptoms [8]. In the early stage of treatment, improvement in pain symptoms can predict higher antidepressant efficacy and a better remission rate [9]. A number of studies have reported that insomnia symptoms were independent predictors for MDD [10, 11], as well as for suicidal ideation and suicide attempts [12, 13]. Another group found a strong correlation between fatigue and depressive symptoms [14], while others showed that fatigue could be used to predict treatment efficacy and future fluctuations in patient well-being [15]. Unexplained breathing difficulties and gastrointestinal disturbances (such as poor appetite and bloating) are also prevalent in MDD, which may cause severe discomfort and result in primary health care visits. However, the pathogenesis of SD is still not fully understood, and it would be interesting and meaningful to explore the mechanisms underlying this condition.

In recent decades, researchers have used noninvasive functional neuroimaging techniques to show that SD is associated with a wide array of frontal, temporal, and parietal cortices [16–18]. Geng et al. found that patients with SD exhibited abnormal regional homogeneity in the frontal and temporal regions [16]. Yan et al. reported that subjects with SD exhibited abnormal amplitude of low frequency fluctuations in the right inferior temporal gyrus, left hippocampus, right inferior frontal orbital gyrus, and left thalamus [18]. Some studies have explored relationships between brain areas and somatic symptoms. For example, pain has been associated with the primary and secondary somatosensory cortices [19], the cingulate [20], and the insula [21], which belong to the so-called “pain matrix” [22]. Insomnia has been associated with impaired activity in the amygdala, thalamus, and insula [23, 24], while fatigue has been associated with impaired connectivity in the default mode network (DMN) [25] and shown to be related to decreased volumes in the prefrontal and occipital cortices [26]. Somatic symptoms include impairments in sensory, cognitive and, most importantly, affective components, which include feelings of sadness, anxiety, and depression in response to noxious stimuli [27]. Although there is no identifiable noxious stimulus in most SD patients, somatic symptoms can occur in the absence of nociceptive input in these individuals [28]. Moreover, somatic symptoms are associated with the severity of emotional symptoms [29]. Identifying which brain regions jointly regulate somatic symptoms and emotion may open new avenues into understanding the pathological mechanisms underlying SD.

The endosensory disorder hypothesis proposed by Harshaw et al. posits that the somatic symptoms of depression are related to an imbalance of internal and external perceptual functions [30]. The insula is an important brain area relevant to internal sensory functions that are responsible for processing sensory afferent information and integrating with higher cortices [31, 32]. Increasing attention has been given to the role of abnormal internal perceptual function of the insula in the pathogenesis of somatic symptoms in MDD [33–35]. The insula is located in the deep part of the bilateral temporal lobes and adjacent to the parietal and occipital lobes [36]. Based on the origins of the cells, the insula can be roughly divided into two subregions with functions that include sensory processing, representing feelings and emotions, motion control, body and self-awareness, risk prediction, and decision-making [37]. The anterior insula (AI) is associated with subcortical brain areas, including the anterior cingulate, ventral medial prefrontal lobe, amygdala, and ventral striatum, that integrate sensory information and participate in emotional, motivational, and cognitive functions [32, 37]. The posterior insula (PI) receives afferent information from the spinal cord and brainstem and participates in processing primary sensory information related to somatosensation and motor control [38, 39]. The AI can also be subdivided into ventral and dorsal parts: the cellular structure of the vAI is more similar to that of the marginal cortex, which may be closely related to emotion, general attention, and cognition; the AI is connected to brain areas related to the cognitive control network and may be involved more in decision-making and behaviour [40]. Zhang et al. found that weaker vAI-right orbitofrontal cortex functional connectivity (FC) strength predicted greater symptom severity [41]. Kandilarova et al. found support for the role of the right AI in the pathophysiology of MDD and found that MDD patients had significantly reduced effective connectivity strength from the AI to the dorsolateral prefrontal cortex, as well as a significant level of effective connectivity strength between the amygdala and the AI [42]. In addition, Stoyanov et al. reported that reduced effective connectivity from the AI to the dorsolateral prefrontal cortex was manifested in patients with depressive symptoms in comparison to patients with paranoid symptoms of schizophrenia [43]. These results indicated that altered effective connectivity in the AI could be a potential diagnostic biomarker to differentiate depressive symptoms [44]. Moreover, as a key node of the salience network (SN), the AI is closely related to the integration of sensory information, emotion, and cognition [45]. Thus, we suggest that abnormal FC between the insular subregions and other brain regions may lead to abnormal somatosensory perception and somatic symptoms [30]. However, to the best of our knowledge, we are not aware of any studies that have examined ventral and dorsal AI and PI FC in SD patients.

To examine how vAI, dAI, and PI affect the corresponding brain circuits in SD, this study used FC analyse of resting-state functional magnetic resonance imaging (rs-fMRI) data. FC measures the correlation coefficients of a single predefined region with another region [46], and these values provide novel insights into how distributed brain regions are functionally integrated in MDD patients [47]. We hypothesized that there would be different patterns of FC damage in different insular subregions. We also hypothesized that abnormal FC in insular subregions might be related to SD severity.

## Methods

### Study design

This study adopted a cross-sectional case-control study design. Data, which included demographic data, clinical assessments and MRI scan data, were collected from patients diagnosed with MDD and healthy controls (HCs).

### Participants

Patients diagnosed with MDD were recruited from the Department of Psychiatry of the Affiliated Nanjing Brain Hospital of Nanjing Medical University between September 2011 and February 2017. The inclusion criteria for this population were as follows: (1) confirmation of the diagnosis of MDD according to DSM-IV-TR criteria [2], (2) age between 18 and 55 years old, (3) right-handed, (4) Chinese Han, (5) regardless of sex, (6) education level of junior high school or above, (7) Hamilton Rating Scale for Depression 17-item (HAMD_17_) [48] score > 17 on the day of scanning, and (8) 32-item hypomania checklist score < 14 [49], and total Young Mania Rating Scale (YMRS) score < 10 [50]. The exclusion criteria in this study were as follows: (1) psychopathologies other than MDD; (2) substance abuse/dependence within the previous 1 year; (3) history of neurological or systemic illness, head injury, or any other relevant medical or additional psychiatric disease; or (4) current pregnancy or breastfeeding.

The patients with MDD were divided into SD and nonsomatic depression (NSD) groups in accordance with previously described criteria [51]. MDD patients were assigned to the SD group when they had three or more of the following symptoms: sleep disturbance, eating disturbance, fatigue, headaches or pain, unexplained breathing difficulty, and poor body image. The patients who had two or fewer abovementioned somatic symptoms were assigned to the NSD group. The inclusion and exclusion criteria for the SD and NSD groups were the same as those we reported in another study [16]. Finally, the study included 28 patients with SD (mean age ± standard deviation = 37.21 ± 7.16 years old; mean education ± standard deviation = 13.36 ± 3.83 years; 15 females) and 30 patients with NSD (mean age ± standard deviation = 34.10 ± 9.91 years old; mean education ± standard deviation = 14.77 ± 2.94 years; 16 females).

Meanwhile, 30 HCs matched in age and sex (mean age ± standard deviation = 35.83 ± 8.15 years old; mean education ± standard deviation = 14.23 ± 2.47 years; 13 females) were recruited from the community with online and flier advertisements. Each HC was screened using the nonpatient version of the Structured Clinical Interview from the DSM-IV-TR [2], and none had any medical illness, neurological illness, psychiatric illness, or a family history of major psychiatric or neurological illness.

Written informed consent was obtained after a complete description of the study was given to all the participants. This study was approved by the Research Ethics Review Board of the Affiliated Nanjing Brain Hospital of Nanjing Medical University.

### Clinical assessments

A homemade questionnaire was used to collect general subject information, including age, sex, years of education, age of onset of depression, number of depressive episodes, and treatment history. The HAMD_17_ and YMRS were used to assess depressive and manic symptom severity, respectively.

### MRI scan acquisitions

Imaging data were obtained from 3-Tesla MRI scans performed at the Affiliated Nanjing Brain Hospital of Nanjing Medical University. The parameters for T1 anatomic axial imaging and echo-planar imaging were the same as those used in our previous articles [16, 18].

### Data preprocessing

Analysis of the functional imaging data was performed using the Data Processing Assistant for Resting-State fMRI (DPARSF) (http://rfmri.org/DPARSF) [52]. The first six functional volumes were discarded to allow the participants to adapt to the scanner noise. Then, slice timing, head-motion correction, and spatial normalization to the Montreal Neurological Institute (MNI) space were conducted. The resampled voxel size was 3 × 3 × 3 mm^3^. These steps were the same as those in our previous research [18]. To ensure data reliability, data were included if subject head movement was < 2 mm with head rotation angle < 2 degrees. We removed several sources of variances that included head-motion parameters, averaged global blood oxygen level-dependent (BOLD) signals and mean BOLD signals in the ventricular and white matter regions. An estimate of head motion at each time point was calculated as the framewise displacement using six displacements from the rigid body-motion correction procedure [53]. The structural images were normalized to the structural (T1-weighted) MNI template. No participant was excluded due to excessive head motion or bad normalization. Then, the remaining data were smoothed using a 4-mm full-width at half maximum (FWHM) Gaussian kernel. Finally, linear detrending and temporal filtering (0.01–0.08 Hz) were performed to reduce low-frequency drift and high-frequency noise.

### FC analysis

We used a seed-based approach to examine FC. First, we defined the bilateral vAI, bilateral dAI, and bilateral PI as regions of interest (ROIs). The centres of each ROI coordinate were derived from the MNI space and included the left vAI (− 33, 13, − 7), right vAI (32, 10, − 6), left dAI (− 38, 6, 2), right dAI (35, 7, 3), left PI (− 38, − 6, 5), and right PI (35, − 11, 6) [40]. Second, we used the centre of each ROI to draw a sphere with a 6-mm radius as an ROI. Third, we employed a voxelwise approach to calculate the correlation between the averaged time series of all voxels in the ROI seeds and the time series from the entire brain template. The correlation coefficients were defined as FC [46] and were converted to z values using Fisher’s transformation to improve normality.

### Statistical analyses

One-way analysis of variance (ANOVA) was used to compare the age, education, total illness duration, and HAMD_17_ scores, while chi-square tests were used to compare the sex ratio in the SD, NSD and HC groups. The disease duration and HAMD_17_ factor scores were compared between the SD and NSD groups using two-sample t tests (SPSS 19.0 software, IBM Corp., Armonk, NY, USA). The level of statistical significance was set at *p* < 0.05 (two-tailed).

Here, we took a two-step approach. First, to compare FC values among the SD, NSD and HC groups, a one-way ANOVA model was applied to six seed-based voxelwise correlation maps using rs-fMRI data analysis toolkit (REST) software [54]. We also regressed out age, sex, and years of education as covariates using regression analysis when performing ANOVA. To control for multiple comparisons, the AlphaSim correction in the REST software was used to adjust the alpha level (4-mm FWHM, individual voxel *p* < 0.001, and minimum cluster size of 162 mm^3^) [54]. There were six cluster maps, and each map showed significant FC values. Second, for each cluster map, post hoc t tests were performed to identify differences between each pair of groups. We continued to use AlphaSim correction to adjust the alpha level (individual voxel *p* < 0.001, and minimum cluster size of 108 mm^3^) with the REST software [54].

Pearson correlation analysis was conducted to explore the possible clinical relevance of the relationship between FC alterations and SD severity. We defined the regions with significant differences in FC between the SD and NSD groups as ROIs. Then, the mean z values of the ROIs were calculated for each SD patient. Finally, Pearson correlation analyses were performed to assess abnormal mean z values of ROIs, total HAMD_17_ scores, and five separate symptomatic factors_,_ including anxiety, weight loss, cognitive disturbance, retardation, and sleep disturbance in HAMD_17_ [48]. To control for multiple comparisons, Bonferroni correction was performed to adjust the alpha level, where the threshold was set to alpha = 0.05/6.

## Results

### Demographics and clinical characteristics

Fifty-eight MDD patients were enrolled in this study, including 28 in the SD group and 30 in the NSD group. Thirty HC subjects were also recruited. SPSS 19.0 software (IBM Corp., Armonk, NY, USA) was used to compare sociodemographic data. There were no statistically significant differences in sex, age, or years of education among the three groups. There was no significant difference in the disease course between the two patient groups. The anxiety/somatization factor scores on the HAMD_17_ in the SD group were significantly higher than those in the NSD group (t = 2.359, *p* = 0.022). The total scores and other factor scores on the HAMD_17_ were not significantly different between the two patient groups (*p* > 0.05); see details in Table 1.

**Table 1:** Subjects’ demographic and clinical characteristics

Variables	SD(*n* = 28)	NSD(*n* = 30)	HC(*n* = 30)	*T/F/χ*	*p*
Sex(M/F)	13/15	14/16	17/13	0.810^a^	0.667^a^
Age, y	37.21 ± 7.16	34.10 ± 9.91	35.83 ± 8.15	0.256^b^	0.775^b^
Education, y	13.36 ± 3.83	14.77 ± 2.94	14.23 ± 2.47	1.158^b^	0.228^b^
Duration of illness, mo	30.64 ± 47.03	68.07 ± 86.23	–	-2.070^c^	0.044^c^
Age at onset, y	34.54 ± 8.52	28.33 ± 8.54	–	2.020^c^	0.048^c^
Depression, No. of episodes	1.64 ± 1.25	2.37 ± 1.71	–	-1.826^c^	0.073^c^
HAMD-17 Total score	23.61 ± 4.13	22.80 ± 4.98	–	0.669^c^	0.506^c^
Anxiety	6.71 ± 2.12	5.37 ± 2.22	–	2.263^c^	0.022^c^
Cognitive disturbance	3.00 ± 1.72	4.00 ± 2.23	–	-1.903^c^	0.062^c^
Retardation	7.75 ± 1.71	8.23 ± 1.87	–	-1.024^c^	0.310^c^
Sleep disturbance	4.32 ± 1.66	4.23 ± 2.11	–	0.176^c^	0.861^c^
Weight loss	0.75 ± 0.97	0.77 ± 0.86	–	-0.070^c^	0.945^c^
Antidepressants					
SSRI	9	14			
SNRI	7	8			
NaSSA	1	3			
Treatment-naive	11	5			

*Abbreviations*: *SD* Somatic depression, *NSD* Non-somatic depression, *HC* Health control, *HAMD* Hamilton Depression Rating Scale

^a^The *χ*/*p* value was obtained by two-tailed Pearson chi-square test

^b^Data were presented as the range of minimum-maximum (mean ± SD). The *F*/*p* value was obtained by one-way analysis of variance

^c^Data were presented as the range of minimum-maximum (mean ± SD). The *T*/*p* value was obtained by two-sample two-tailed *t* test

### Insula-seed FC analysis

**Fig. 1:**
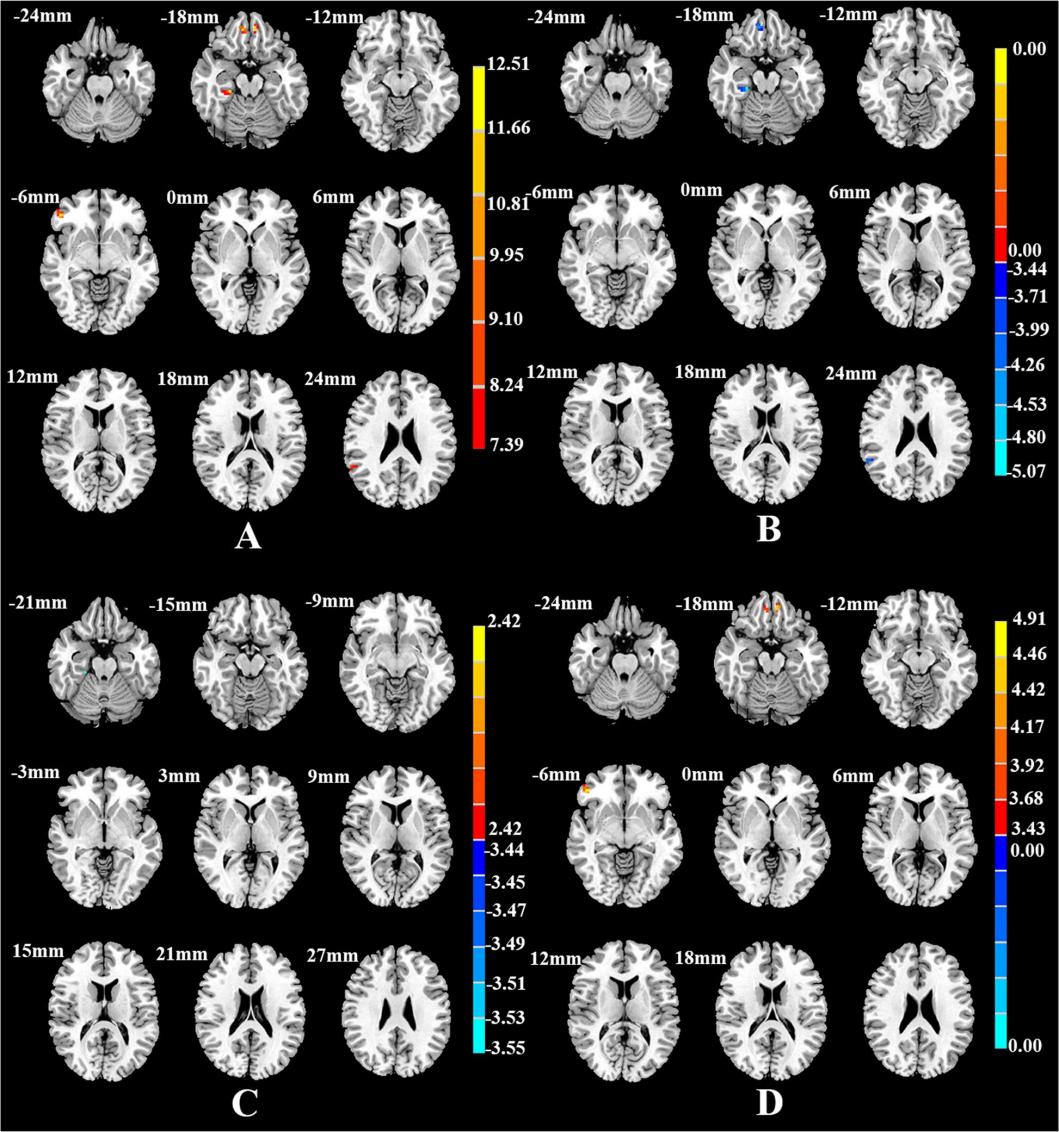
Brain regions showing differences in the left vAI-seed FC among the SD, NSD, and HC groups (**A**), between the SD and NSD groups(**B**), between the SD and HC groups(**C**), and between the NSD and HC groups(**D**). *p* < 0.05, corrected for multiple comparisons using AlphaSim correction

**Fig. 2:**
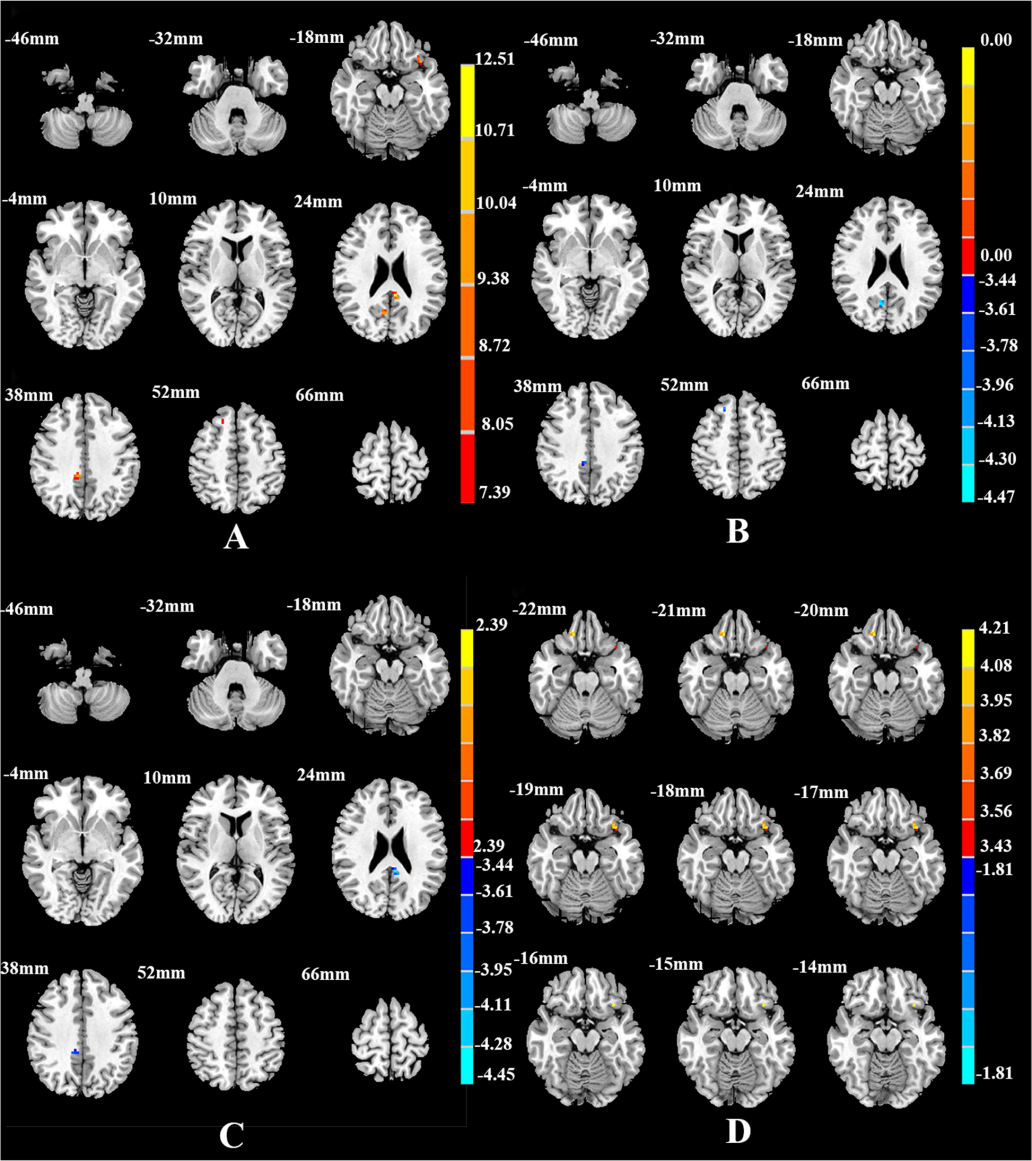
Brain regions showing differences in the right vAI-seed FC among the SD, NSD, and HC groups (**A**), between the SD and NSD groups(**B**), between the SD and HC groups(**C**), and between the NSD and HC groups(**D**). *p* < 0.05, corrected for multiple comparisons using AlphaSim correction

**Fig. 3:**
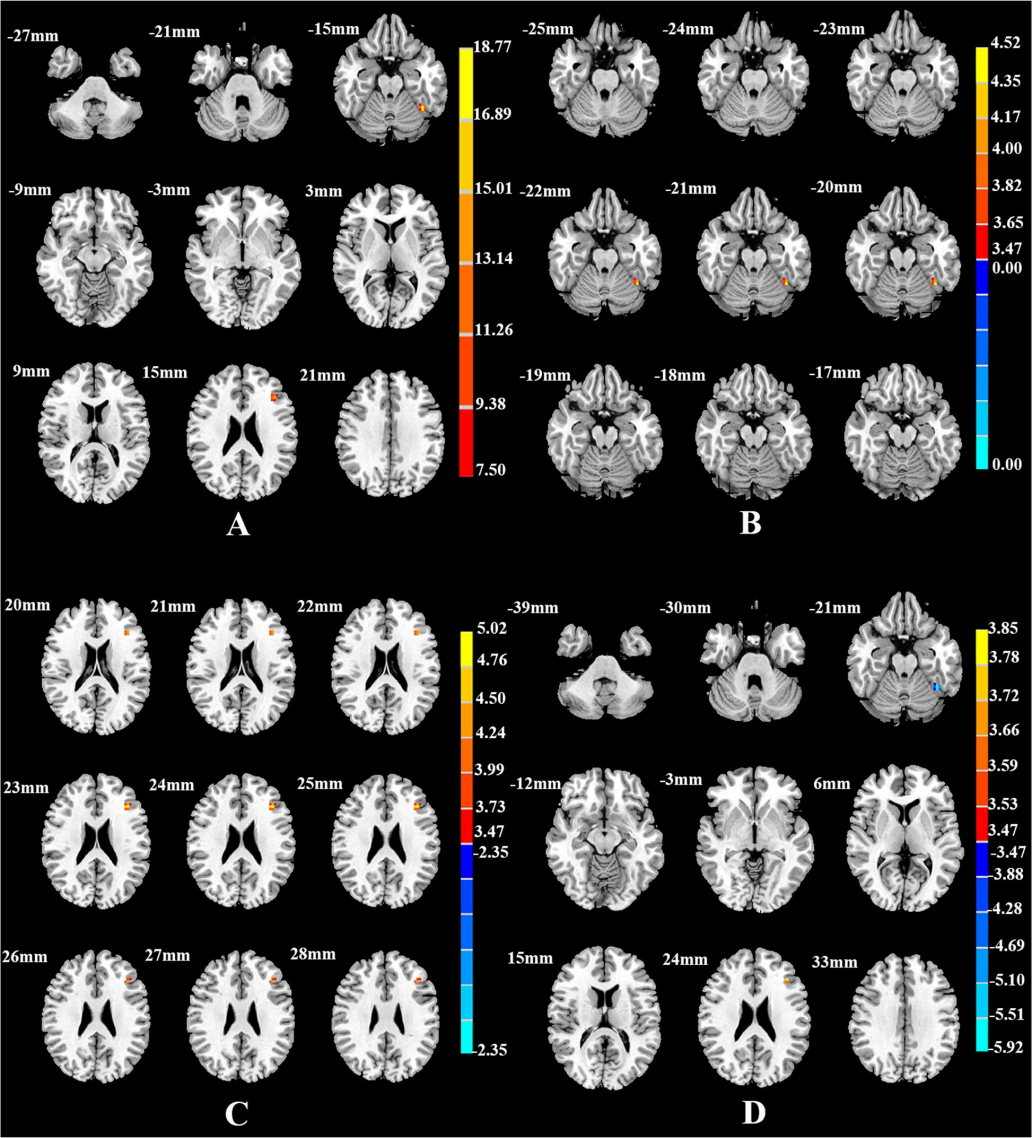
Brain regions showing differences in the left dAI-seed FC among the SD, NSD, and HC groups (**A**), between the SD and NSD groups(**B**), between the SD and HC groups(**C**), and between the NSD and HC groups(**D**). *p* < 0.05, corrected for multiple comparisons using AlphaSim correction

**Fig. 4:**
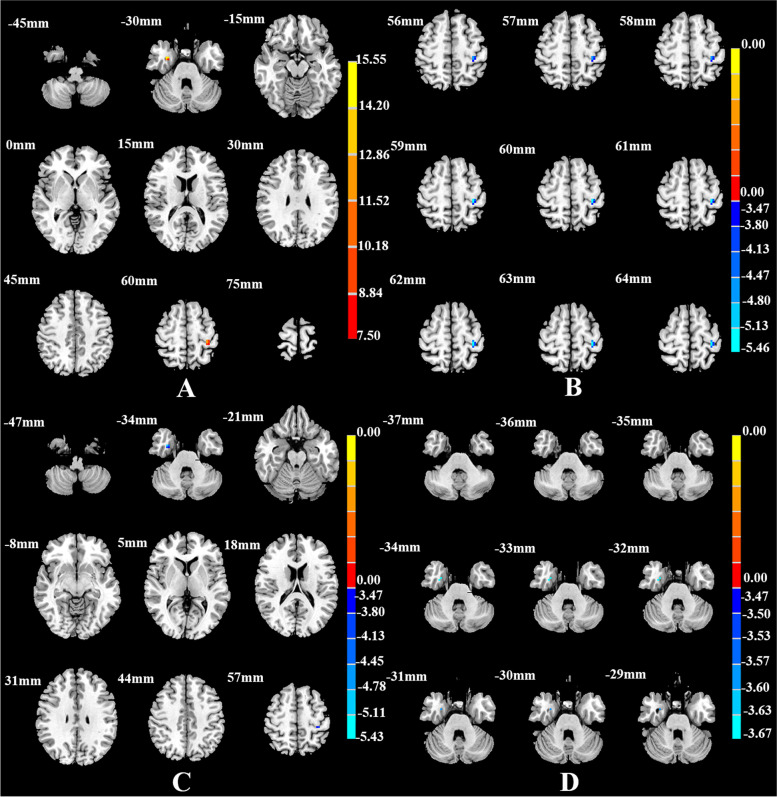
Brain regions showing differences in the right dAI-seed FC among the SD, NSD, and HC groups (**A**), between the SD and NSD groups(**B**), between the SD and HC groups(**C**), and between the NSD and HC groups(**D**). *p* < 0.05, corrected for multiple comparisons using AlphaSim correction

Among the three groups, there were FC differences between the left vAI and the bilateral rectus gyri, bilateral fusiform gyri, right orbital inferior frontal gyrus, and right angular gyrus (Table 2, Fig. 1A); between the right vAI and the bilateral orbital frontal gyri, right superior frontal gyrus, right middle cingulate cortex, and right precuneus (Table 3, Fig. 2A); between the left dAI and the left fusiform gyrus and left inferior frontal gyrus (Table 4, Fig. 3A); and between the right dAI and the right fusiform gyrus and left postcentral gyrus (Table 5, Fig. 4A) (*p* < 0.001, k > 6 voxels, *p* < 0.05 corrected for multiple comparisons using AlphaSim).

**Table 2:** Brain areas with significantly different FC among the SD, NSD, and HC groups using the left vAI as the seed region

Brain regions	MNI (x y z)	Cluster size	F*/t* -value
Three groups			
Rectus_L	-6 48–18	9	11.258^a^
Rectus_R	9 45–18	11	11.135^a^
Fusiform_R	24–30 -18	10	12.893^a^
Inferior frontal orbital gyrus_R	48 39–6	23	11.666^a^
Angular_R	63–51 27	9	8.885^a^
SD vs NSD			
Rectus_R	9 45–18	10	−4.513^b^
Fusiform_R	24–30 -18	10	-5.146^b^
Angular_R	60–51 24	9	-4.341^b^
SD vs HC			
Fusiform_R	24–30 -21	4	-4.493^b^
NSD vs HC			
Rectus_L	−6 48–18	6	4.002^b^
Rectus_R	3 42–21	9	4.227^b^
Inferior frontal orbital gyrus_R	48 39–9	20	4.575^b^

*SD* Somatic depression, *NSD* Non-somatic depression, *HC* Healthy control, *vAI* ventral anterior insula, *MNI* Montreal Neurological Institute, x, y, z are the coordinates of the primary peak locations in the MNI space; F is obtained by one-way analysis of variance; *t* is obtained by two-sample two-tailed *t* test (*p* < 0.05, alphasim correction), *L* Left, *R* Right

^a^The F statistical value

^b^The t statistical value

**Table 3:** Brain areas with significantly different FC among the SD, NSD, and HC groups using the right vAI as the seed region

Brain regions	MNI (x y z)	Cluster size	*F/t* -value
Three groups			
Superior frontal orbital gyrus_R	15 36–21	6	9.581^a^
Inferior frontal orbital gyrus_L	−36 18–18	7	9.403^a^
Superior frontal gyrus_R	21 27 54	7	8.568^a^
Middle cingulate gyrus_R	9–39 33	20	11.745^a^
Posterior cingulate gyrus_L	−6 − 42 24	7	11.388^a^
Precuneus_R	9–63 24	9	9.456^a^
SD vs NSD			
Middle cingulate gyrus_R	9–39 33	16	−4.657^b^
Precuneus_R	9–63 24	9	− 4.483^b^
Superior frontal gyrus_R	21 27 54	7	-4.190^b^
SD vs HC			
Posterior cingulate gyrus_L	−6 − 42 24	7	−4.717^b^
Precuneus_R	12–39 39	16	-4.722^b^
NSD vs HC			
Superior frontal orbital gyrus_R	18 36–21	6	3.852^b^
Inferior frontal orbital gyrus_L	−33 21–18	3	3.588^b^

*SD* Somatic depression, *NSD* Non-somatic depression, *HC* Healthy control, *vAI* Ventral anterior insula, *MNI* Montreal Neurological Institute, x, y, z are the coordinates of the primary peak locations in the MNI space; *F* is obtained by one-way analysis of variance; *t* is obtained by two-sample two-tailed *t* test (*p* < 0.05, alphasim correction); *L* left, *R* Right

^a^The *F* statistical value

^b^The *t* statistical value

**Table 4:** Brain areas with significantly different FC among the SD, NSD, and HC groups using the left dAI as the seed region

Brain regions	MNI (x y z)	Cluster size	*F/t* -value
Three groups			
Fusiform_L	−39 -51 -21	9	18.625^a^
Inferior frontal gyrus_L	−39 27 24	19	13.632^a^
SD vs NSD			
Fusiform_L	−39 -54 -21	5	4.522^b^
SD vs HC			
Inferior frontal gyrus_L	−39 27 24	16	5.017^b^
NSD vs HC			
Fusiform_L	−39 − 51 -21	9	-5.921^b^
Inferior frontal gyrus_L	−39 30 24	7	3.848^b^

*SD* Somatic depression, *NSD* Non-somatic depression, *HC* Healthy control, *dAI* Dorsal anterior insula, *MNI* Montreal Neurological Institute, x, y, z are the coordinates of the primary peak locations in the MNI space; *F* is obtained by one-way analysis of variance; *t* is obtained by two-sample two-tailed *t* test (*p* < 0.05, alphasim correction); *L* Left, *R* Right

^a^The *F* statistical value

^b^The *t* statistical value

**Table 5:** Brain areas with significantly different FC among the SD, NSD, and HC groups using the right dAI as the seed region

Brain regions	MNI (x y z)	Cluster size	F*/t* -value
Three groups			
Fusiform_R	33–3 -30	8	15.960^a^
Postcentral_L	−33–33 60	19	12.451^a^
SD vs NSD			
Postcentral_L	−33–30 63	17	−5.457^b^
SD vs HC			
Fusiform_R	33–3 -30	8	−5.433^b^
Postcentral_L	−36 − 33 57	7	−3.807^b^
NSD vs HC			
Fusiform_R	30–3 -33	3	-3.669^b^

*SD* Somatic depression, *NSD* Non-somatic depression, *HC* Healthy control, *dAI* Dorsal anterior insula, *MNI* Montreal Neurological Institute, x, y, z are the coordinates of the primary peak locations in the MNI space; F is obtained by one-way analysis of variance; t is obtained by two-sample two-tailed *t* test (*p* < 0.05, alphasim correction); *L* left, *R* Right

^a^The F statistical value

^b^The t statistical value

Compared with the NSD group, the SD group showed lower FC between the AI subregions and the frontal and limbic cortices. FC was lower between the left vAI and the right rectus, right fusiform gyrus, and right angular gyrus (Table 2, Fig. 1B); between the right vAI and the right middle cingulate cortex, right precuneus, and right superior frontal gyrus (Table 3, Fig. 2B); between the left dAI and the left fusiform gyrus (Table 4, Fig. 3B); and between the right dAI and the left postcentral gyrus (Table 5, Fig. 4B) (*p* < 0.001, k > 6 voxels, *p* < 0.05 corrected for multiple comparisons using AlphaSim).

Compared with the HC group, the SD and NSD groups exhibited very different alterations in AI functional coupling, with lower FC in the SD patients but higher FC in the NSD patients. The SD group showed lower FC between the left vAI with the right fusiform gyrus (Table 2, Fig. 1C), between the right vAI with the left posterior cingulate cortex and right precuneus (Table 3, Fig. 2C), between the left dAI with the left inferior frontal gyrus (Table 4, Fig. 3C), and between the right dAI with the right fusiform gyrus and left postcentral gyrus (Table 5, Fig. 4C). The NSD group showed higher FC between the left vAI with the bilateral rectus gyri and right orbital inferior frontal gyrus (Table 2, Fig. 1D), between the right vAI with the bilateral orbital frontal gyri (Table 3, Fig. 2D), between the left dAI with the left fusiform gyrus and left inferior frontal gyrus (Table 4, Fig. 3D), and between the right dAI with the right fusiform gyrus (Table 5, Fig. 4D) (*p* < 0.001, k > 6 voxels, *p* < 0.05 corrected for multiple comparisons using AlphaSim).

There were no differences in FC between the bilateral PI and any brain regions among the SD, NSD, and HC groups.

### Exploratory correlational analyses between FC values and clinical data in SD patients

Our study did not find a significant correlation between FC values and any clinical characteristics in the SD patients. Some correlations failed to survive the multiple comparison correction. In the SD group, FC values between the left vAI and right rectus gyrus were positively correlated with cognitive impairment factor scores on the HAMD_17_ (*r* = 0.35, *p* = 0.040, uncorrected), and FC values between the right vAI and right superior frontal gyrus were positively correlated with total HAMD_17_ scores and cognitive impairment factor scores (*r* = 0.42 and *p* = 0.015, *r* = 0.43 and *p* = 0.013, uncorrected).

## Discussion

The present study applied resting-state FC analyses to explore functional synchronization between the insula and the whole brain in MDD patients with somatic symptoms. The results showed that these subjects had decreased FC between the vAI and dAI and right frontal regions (superior frontal and rectus gyri) and several limbic brain areas (precuneus, angular gyrus, fusiform gyrus, and middle cingulate cortex). Unfortunately, subsequent Pearson correlation analyses revealed that there were no significant correlations between averaged FC values and some clinical indices. The uncorrected results showed that FC values between the left vAI and right rectus gyrus positively correlated with the cognitive impairment factor scores on the HAMD_17_ in the SD patients, while FC values between the right vAI and right superior frontal gyrus were positively correlated with the total and cognitive impairment factor scores on the HAMD_17_ in the SD patients. Patients without somatic symptoms (NSD group) showed enhanced FC between the AI and bilateral orbitofrontal cortices, rectus gyri and fusiform gyri.

The insula can be roughly divided into anterior and posterior subregions based on different cell origins and functions [31, 55]. The PI receives interoceptive signals from the spinal cord and brainstem and participates in the processing of primary sensory information such as somatosensory and motor control. The AI is connected with several regions in the frontal cortex, including the anterior cingulate cortex, medial prefrontal cortex, amygdala, and ventral striatum; it is involved in the processes of emotion, motivation, and cognition [55, 56]. The posterior-to-anterior transmission of interoceptive signals and interactions with multiple other brain regions enables conscious emotional perception of interoceptive information [31]. Previous functional neuroimaging work demonstrated a cognition - emotion - interoception division of the insula [56], dividing the AI into the ventral and dorsal subregions. The vAI has a similar cell structure as the limbic cortex, suggesting that it is more involved in emotion and attention, while the dAI is connected to frontal areas that function in cognitive control [40, 57]. With regard to large-scale networks, the insula is an important component of the salience network (SN), which is responsible for switching between the central executive network (CEN; task activated) and the DMN (task-negative activated); this structure comprehensively evaluates the importance of internal and external stimuli to affect cognitive emotional behaviour [58]. According to a recent review [37], patients with depression have abnormalities in the structure, activation, and FC of the insula, which may help guide the treatment of depression.

Our results showed decreased FC between the vAI and dAI with right frontal regions (superior frontal gyrus and rectus gyrus) in the SD group. Similar findings have been reported in previous studies. Zhang et al. investigated resting-state FC based on ventral and dorsal AI seeds; they concluded that depressive patients had increased FC between the vAI and orbitofrontal cortex, and FC values were negatively correlated with somatic symptom severity. Moreover, these FC decreases could be reversed with electroconvulsive therapy [35]. Yao et al. found that insular activity and FC between the insula and prefrontal cortex were reduced in patients with abnormal pain empathy [59]. The intervention measures improved pain empathy and increased FC between the insula and prefrontal cortex [59], suggesting that the decrease in functional coupling between these structures may be related to abnormal somatic perception, which is consistent with our observation of lowered insula-prefrontal cortex FC in depressed patients with somatic symptoms. Kim et al. demonstrated that patients with complex regional pain syndrome showed decreased FC between the AI and the dorsomedial prefrontal cortex, inferior frontal gyrus and cingulate cortex [60]. The superior frontal gyrus plays an important role in self-awareness, emotion regulation, and cognitive processing [61, 62]. The rectus gyrus is a part of the medial prefrontal cortex that mediates sensory information integration by connecting the prefrontal cortex with the hypothalamus and brainstem [63]. It was reported that deep brain stimulation therapy targeting the rectus gyrus can effectively improve refractory depression [64]. In addition, the superior frontal gyrus is a key region of the CEN [65] that participates in cognitive control and decision-making [66]. The insula is an important component of the SN. Decreased FC between the AI and superior frontal gyrus in depressed patients may reflect abnormal functional regulation between the SN and CEN, thus contributing to somatic symptoms. The trends in the correlations between FC values between the left vAI-right rectus gyrus and right vAI-right superior frontal gyrus with cognitive factors in the present study suggest that abnormal coupling of the AI and frontal cortex might induce somatic symptoms by affecting the cognition of patients with MDD. Compared with the HC group, the NSD group showed enhanced FC between the AI and the bilateral orbitofrontal cortices, rectus gyri, and fusiform gyri. The opposite patterns of AI connectivity in the two depression subgroups indicated that functional synchronicity between the AI and the abovementioned brain regions may play important roles in the somatic symptoms of depression. We speculated that functional synchronization between the insula and superior frontal gyrus and the rectus was weakened, inducing abnormal somatosensory information processing that resulted in uncomfortable somatic symptoms.

We also found decreased FC between the AI (especially the ventral AI) and several limbic brain regions (precuneus, angular gyrus, fusiform gyrus and middle cingulate cortex). These results were consistent with the aforementioned different FC patterns of the vAI and dAI with other brain regions and confirm the close interactions between the vAI and the limbic system. Considering that the limbic system is closely related to emotion and attention, aberrant FC between the vAI and limbic cortex may influence somatic and visceral sensory processes, as well as autonomic regulation of the heart and gastrointestinal tract. Avery et al. reported decreased activity in the dorsal mid-insula cortex (dmIC) during a task requiring attention to visceral interoceptive sensations, as well as increased resting-state FC between the dmIC and limbic brain regions that was positively correlated with depression severity and somatic symptoms [67]. On the other hand, the precuneus, superior marginal gyrus, and angular gyrus are important components of the DMN that are involved in emotional cognition and executive functions such as motivation, memory, and self-referential activity. The insula is a core node of the SN. Decreased functional coupling between the AI and the abovementioned limbic areas probably indicates inadequate regulation between the SN and DMN [68], leading to the presentation of somatic symptoms in MDD.

There were no differences in FC between the bilateral PI and any other brain regions in the depressive patients with or without somatic symptoms and HCs. The PI is responsible for the preliminary processing of sensory information, and is a critical hub for central integration and processing of painful stimuli [69]. Studies have found that the PI was associated with interoceptive awareness and relieved patients from the sensory and affective burden of chronic pain. Moreover, disruption functional connectivity of right PI were found in MDD [69–71]. Our results suggest that the somatic symptoms of patients with MDD mainly involve abnormal cognitive regulation and not primary sensory information processing [72]. Regardless of the important role of the PI in the primary processing of interoceptive signals, fMRI results in previous studies have been inconsistent. Gu et al. claimed that activity in the bilateral AI but not the PI was aberrant during tasks assessing cognitive demand and pain stimulus valence. Importantly, these cognitive demand and stimulus valence tasks resulted in a significant interaction in the AI and regions of the frontoparietal network [73]. Conversely, decreased FC between both the AI and PI and the frontal and cingulate cortices was reported in patients with pain syndrome [60]. Such discrepancies may be due to different diseases and task paradigms. The obvious difference in FC of the AI and PI underscores the importance of researching insular subregions.

### Limitations

There are some limitations that should be considered in the present study. First, we defined seeds with population-level but not individual-level atlases, which may have concealed actual individual variations in brain functioning. In addition, as a real-world study, medication was not restricted before admission to this study, and a number of patients were prescribed antidepressants. Although electroconvulsive therapy was reported to reverse abnormal insular FC [35], it is unclear whether medication influenced insular FC in the present study. Therefore, we used drugs as covariates to reduce the impact of drugs on result. Additionally, the correlation between FC values and clinical scale scores failed to survive after corrections for multiple comparisons. This may have been due to the small sample size, which limited the generalization of our findings. Finally, we used the FC method to calculate FC values of the insula to the whole brain. However, unlike the effective connectivity method, FC has no directionality [44]. Thus, future studies could utilize an effective connectivity approach in a larger sample size of medication-free patients to further explore causality as it relates to FC.

## Conclusion

In summary, the present study revealed aberrant functional synchronization between the AI and frontal and limbic cortices. These changes indicate abnormal interactions between the three main large-scale brain functional networks (SN, DMN, and CEN) that might underlie somatic symptoms in SD by affecting the cognitive and emotional processing of interoceptive information.

## Supplementary Information

**Additional file 1.**

## Data Availability

The datasets generated during the current study are not publicly available due to the subjects’ privacy, but are available from the corresponding author on reasonable request.

## Abbreviations

MDD: Major depressive disorder
SD: Somatic depression
NSD: Non-somatic depression
FC: Functional Connectivity
vAI: Ventral anterior insula
dAI: Dorsal anterior insula
PI: Posterior insula
dmIC: dorsal mid-insula cortex
HAMD_17_: 17-item Hamilton Rating Scale for Depression
DMN: Default mode network
SN: Salience network
YMRS: Young Mania Rating Scale
HCs: Healthy controls
DSM-IV-TR: Statistical Manual of Mental Disorders, 4th Edition, Text Revision
DPARSF: Data Processing Assistant for Resting-State fMRI
MNI: Montreal Neurological Institute
BOLD: Blood oxygen-level dependent
FWHM: Full-width at half maximum
ROIs: Regions of interest
REST: Rs-fMRI data analysis toolkit
