# Supplementary material for: Aberrant functional connectivity in insular subregions in somatic depression: a resting-state fMRI study
Source: BMC Psychiatry. 2022 Feb 24;22:146. doi: 10.1186/s12888-022-03795-5 (PMC8867834; doi:10.1186/s12888-022-03795-5)
Supplement: Supplementary file 1 — Additional file 1. [file 12888_2022_3795_MOESM1_ESM.doc]

Supplement Table 1

Correlational analyses between the differences FCs values and the clinical data in SD

| FCs values | HAMD-17  (*r*/*p*) | Anxiety (*r*/*p*) | Cognitive disturbance  (*r*/*p*) | Retardation  (*r*/*p*) | Sleep disturbance  (*r*/*p*) | Weight loss  (*r*/*p*) |
| --- | --- | --- | --- | --- | --- | --- |
| vAI_L-Rectus_R | 0.064/0.745 | 0.066/0.739 | -0.027/0.890 | 0.351/0.040 | 0.211/0.282 | 0.098/0.621 |
| vAI_L-Fusiform_R | 0.043/0.827 | 0.003/0.986 | -.110/.578 | -.043/.829 | .106/.592 | 0.033/0.869 |
| vAI_L-Angular_R | -0.055/0.781 | -0.093/0.639 | -.161/.413 | .019/.924 | .189/.336 | -0.036/0.854 |
| vAI_R-Middle cingulate gyrus_R | 0.144/0.464 | -0.168/0.394 | 0.106/0.593 | -0.224/0.251 | 0.232/0.242 | 0.081/0.681 |
| vAI_R-Precuneus_R | 0.142/0.472 | -0.195/0.321 | 0.139/0.480 | -0.149/0.450 | 0.334/0.061 | 0.234/0.230 |
| vAI_R-Superior frontal gyrus_R | -0.126/0.522 | -0.230/0.238 | -0.020/0.919 | 0.097/0.623 | 0.115/0.561 | -0.300/0.121 |
| dAI_L-Fusiform_L | 0.423/0.015 | 0.052/0.792 | 0.430/.013 | -0.109/0.582 | 0.144/0.464 | -0.078/0.695 |
| dAI_R-Postcentral_L | 0.134/0.497 | 0.178/0.364 | -0.166/0.398 | 0.051/0.797 | -0.016/0.937 | 0.233/0.232 |

vAI, ventral anterior insula; dAI, dorsal anterior insula; HAMD, Hamilton Depression Rating Scale. L = left; R = right.
